# Chimeric Antigen Receptor-Modified T Cell Therapy in Multiple Myeloma: Beyond B Cell Maturation Antigen

**DOI:** 10.3389/fimmu.2019.01613

**Published:** 2019-07-16

**Authors:** Marijke Timmers, Gils Roex, Yuedi Wang, Diana Campillo-Davo, Viggo F. I. Van Tendeloo, Yiwei Chu, Zwi N. Berneman, Feifei Luo, Heleen H. Van Acker, Sébastien Anguille

**Affiliations:** ^1^Division of Hematology, Center for Cell Therapy and Regenerative Medicine, Antwerp University Hospital, Antwerp, Belgium; ^2^Laboratory of Experimental Hematology, Faculty of Medicine & Health Sciences, Vaccine and Infectious Disease Institute, University of Antwerp, Antwerp, Belgium; ^3^Biotherapy Research Center, Fudan University, Shanghai, China; ^4^Department of Digestive Diseases, Huashan Hospital of Fudan University, Shanghai, China

**Keywords:** chimeric antigen receptor-modified T cells, immunotherapy, multiple myeloma, B cell maturation antigen, CD19, CD138, CD38, SLAMF7/CS1

## Abstract

Chimeric antigen receptor (CAR)-modified T cell therapy is a rapidly emerging immunotherapeutic approach that is revolutionizing cancer treatment. The impressive clinical results obtained with CAR-T cell therapy in patients with acute lymphoblastic leukemia and lymphoma have fueled the development of CAR-T cells targeting other malignancies, including multiple myeloma (MM). The field of CAR-T cell therapy for MM is still in its infancy, but remains promising. To date, most studies have been performed with B cell maturation antigen (BCMA)-targeted CARs, for which high response rates have been obtained in early-phase clinical trials. However, responses are usually temporary, and relapses have frequently been observed. One of the major reasons for relapse is the loss or downregulation of BCMA expression following CAR-T therapy. This has fostered a search for alternative target antigens that are expressed on the MM cell surface. In this review, we provide an overview of myeloma target antigens other than BCMA that are currently being evaluated in pre-clinical and clinical studies.

## Introduction

Multiple myeloma (MM) is a malignant neoplasm of plasma cells that accumulates in the bone marrow, leading to bone destruction, and marrow failure. With an incidence of five cases/100,000 individuals/year in Western countries, MM accounts for 1% of all cancers and for ~10% of all hematological malignancies (1). MM arises from a pre-malignant asymptomatic proliferation of plasma cells (monoclonal gammopathy of unknown significance and smoldering MM). These can further evolve into symptomatic MM with end-organ damage, which is associated with significant morbidity (2). Despite the availability of various therapeutic agents, including proteasome inhibitors (e.g., bortezomib), immunomodulatory drugs (e.g., lenalidomide), and monoclonal antibodies (e.g., daratumumab and elotuzumab), the disease remains incurable (3).

**Figure 1:**
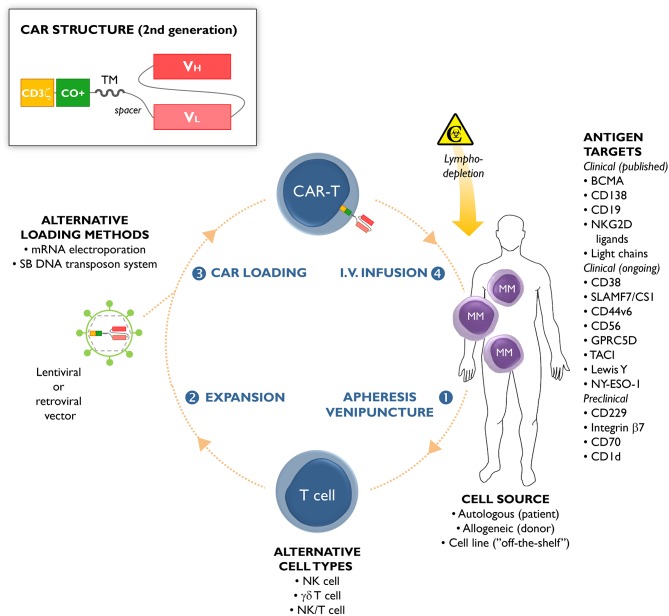
Chimeric antigen receptor (CAR)-T cells from multiple myeloma (MM) patients are usually manufactured from autologous T cells collected through leukapheresis or venipuncture (step 1). Apart from autologous cells, allogeneic cells or cell lines have been used as starting material (11). Natural killer (NK) cells, γδ T cells, and NK/T cells have been used as alternative lymphocyte subsets for CAR-T manufacturing. In a next step, the cells are expanded *ex vivo* (step 2) and loaded (step 3) with a lentiviral or retroviral vector carrying the *CAR* gene. CAR loading can also be accomplished by non-viral methods, including messenger RNA (mRNA) electroporation or using the Sleeping-Beauty (SB) DNA transposon system. The CAR-loaded T cells are administered by intravenous infusion (step 4) to the patient, who has usually received prior lympodepleting chemotherapy (such as cyclophosphamide or fludarabine). The different MM antigens that can serve as targets for CAR-T cell-based immunotherapy are schematically depicted, including their stage of clinical development (published clinical trials, ongoing clinical trials, pre-clinical studies). The insert shows the common structure of a second-generation CAR construct. The extracellular part of a CAR is composed of the antigen-recognition domain from a monoclonal antibody (usually with the V_H_ and V_L_ chains in single-chain variable fragment [scFv] format), and an extracellular spacer. The transmembrane (TM) and intracellular domains are the other CAR constituting parts. The latter contains a costimulatory (CO+) domain (e.g., 4-1BB or CD28), and the CD3ζ chain of the T-cell receptor.

Cellular engineering has provided various opportunities to redirect the immune system against malignant cells. For example, adoptive transfer of chimeric antigen receptor (CAR)-engineered T cells is an emerging therapeutic strategy that has already shown unprecedented results in CD19-expressing hematological malignancies (4–7). These results have spurred new interest in the further development of this technology. The majority of CAR-T cell approaches have been applied to αβ T cells or occasionally natural killer (NK) cells (8), γδ T cells (9), or NK/T cells (10), as the effector cells of choice (Figure 1). The concept behind this therapy is that CAR-engineered immune cells and their effector functions are redirected against malignant cells bearing the antigen of interest, irrespective of the patients' human leukocyte antigen (HLA) genetics.

Chimeric antigen receptors comprise (i) an ectodomain binding directly a tumor-specific molecule on the cell surface, (ii) an extracellular hinge/spacer and a transmembrane domain spanning the membrane, and (iii) an endodomain providing T cell signaling (Figure 1). The ectodomain is generally derived from the antigen binding regions of a monoclonal antibody (12). The endodomain is composed of the CD3ζ signaling chain, providing an activation signal termed signal 1. Second- and third-generation CARs have additional costimulatory molecule domains, e.g., CD28, OX40, or 4-1BB (signal 2). Fourth-generation CARs, also known as T cells redirected for universal cytokine-mediated killing, express additional molecules to enhance CAR-T cell efficacy, such as inducible interleukin (IL)-12 (13).

To date, two CD19-specific CAR-T cell products (Kymriah and Yescarta) have been approved by the US Food and Drug Administration and the European Medicines Agency. Although the use of CAR-T cells in the treatment of MM is still confined to a handful of antigens and early-phase clinical trials, CAR-T cell therapy holds the potential to fulfill the unmet medical needs of patients with relapsed/refractory MM.

In multiple myeloma, B-cell maturation antigen (BCMA) is a commonly used target antigen in CAR-T cell clinical trials (14–16). BCMA, also known as tumor necrosis factor receptor superfamily member 17, is highly expressed on malignant plasma cells (17, 18). No expression of BCMA has been observed in normal cells/tissues, except for healthy, differentiated B cells where it is usually expressed at low level. BCMA appears to be a vital in promoting MM cell survival, proliferation, and drug resistance (19, 20) and can be used to monitor the disease course and predict patient outcomes (21).

Table 1 summarizes the clinical outcome of all hitherto published clinical trials of BCMA-targeting CAR-T cell therapies in MM (22–27). BCMA CAR-T cell therapy produces objective response rates of up to 88% (Table 1). Nevertheless, the therapeutic effect is often temporary and relapses are commonly being reported. As shown in Table 1, the median progression-free survival of BCMA CAR-T cell therapy is in the range of 12 months. Downregulation or loss of BCMA expression is likely an important mechanism underlying these relapses (28, 29). Hence, alternatives for BCMA are now under intensive investigation in the field of CAR-T cell therapy for MM (16, 30). The goal of this review is to outline the different target antigens other than BCMA that are currently being evaluated. In the first part, summarized in Table 2, an overview is given of non-BCMA CAR-T cell trials for which (preliminary) results have already been published in Web of Science-listed papers. In the second part, we will focus on alternative target antigens that have entered into CAR-T cell clinical trials. In the third and final part, we will briefly touch upon new antigens that are undergoing pre-clinical evaluation for use in CAR-T cell therapy for MM (schematically depicted in Figure 1).

**Table 1:** Published clinical results of multiple myeloma CAR-T cell clinical trials targeting BCMA.

**CAR-T cell product ^**(ref.)**^**	***n* =**	**ORR (*n* =)**	**median PFS (95% CI)**
bb2121 (22)	33	85% (28)	11.8 months (6.2–n.e.)^$^
CART-BCMA Upenn (23)	25	48% (12)	2.0 months (ND)
NCI CAR BCMA-T (24)^#^	10	20% (2)	1.5 months (ND)
NCI CAR BCMA-T (25)*	16	81% (13)	7.25 months (ND)
LCAR-B38M (26)	17	88% (15)	12.2 months (ND)
LCAR-B38M (27)	57	88% (50)	15.0 months (11.0–n.e.)

Only fully published clinical studies were included (last search: May 1, 2019). (ref.), bibliography reference; n =, number of patients; ORR, objective response rate, defined as the sum of complete responses and (very good) partial responses; PFS, progression-free survival; 95% CI, 95% confidence interval; n.e., not estimable, ND, no data;

$ PFS calculated for 30 patients treated with active doses of bb2121 only (i.e., ≥150 × 10^6^ CAR-T cells); ^#^lower dose cohorts (i.e., 0.3-1-3 × 10^6^ CAR-T cells/kg), *highest dose cohort (i.e., 9 × 10^6^ CAR-T cells/kg).

**Table 2:** Published results of multiple myeloma CAR-T cell clinical trials targeting antigens other than BCMA.

***n* = (ref.)**	**Antigen**	**Signaling domains**	**Cell source/type**	**Transfer method**	**Conditioning**	**T-cell dosage**	**Therapy-related side effects**	**Clinical effects**
*n* = 1 (31)	CD138	ND	Autologous T cells	ND	CP/Flu	1.5 × 10^8^	• CRS gr. 2 (1)	• PR (1)
*n* = 5 (32)	CD138	4-1BB/CD3ζ	Autologous T cells	Lentiviral	PCD, CP or VAD	0.756 × 10^7^/kg	• Infusion-related fever (4)	• SD > 3 m (4)
							• Nausea and vomiting (3)	• ↓ circulating PCL cells (1)
							• ↑ Liver function tests (1)	
							• Possible TLS (1)	
*n* = 10 (33)	CD19	4-1BB/CD3ζ	Autologous T cells	Lentiviral	HDM + ASCT	1–5 × 10^7^	• Hypogammaglobulinemia (1)	• CR (1)
							• Autologous GvHD (1)	• VGPR (6/10) at d100 post-ASCT
							• Mucositis (1)	• PR (2/10) at d100 post-ASCT
*n* = 5/8 (34)	CD19 + BCMA	OX40/CD28	Autologous or allogeneic T cells	Lentiviral	CP/Flu	1 × 10^7^/kg	• CRS gr. 1–2 (7), gr.≥3 (1)	• sCR (1/5)
							• Prolonged cytopenias (5/5)	• VGPR (1/5)
							• Coagulopathy (5)	• PR (2/5)
							• ↑ Liver function tests (4)	• SD (1/5)
							• Pulmonary edema (3)	
							• Pleural effusion and ascites (1)	
*n* = 10 (35)	CD19 + BCMA	OX40/CD28	Autologous T cells	Lentiviral	Bu-CP + ASCT	1 × 10^7^/kg	• CRS gr. 1–2 (10)	• CR (7/10)
							• Coagulopathy (7)	• VGPR (3/10)
							• ↑ Troponin levels (4)	
							• Atrial flutter (1)	
*n* = 5 (36)	NKG2D ligands	CD3ζ	Autologous T cells	Retroviral	None	1–3 × 10^6−7^	• None	• None
*n* = 7 (37)	κLC	CD28/CD3ζ	Autologous T cells	Retroviral	CP (4)	0.92–1.9 × 10^8^/m^2^	• Lymphopenia gr. 3 (1)	• SD 6 wk−24m (4)
					or none (3)			

*Only fully published clinical studies were included (last search: May 1, 2019). n =, number of patients; (ref.), bibliography reference; ASCT, autologous stem cell transplantation; BCMA, B cell maturation antigen; Bu, busulphan; CP, cyclophosphamide; CRS, cytokine release syndrome; d, days; Flu, fludarabine; GvHD, graft-vs.-host disease; HDM, high-dose melphalan; κLC, kappa light chain; m, months; ND, no data; NKG2D, natural killer group 2, member D; PCD, pomalidomide-cyclophosphamide-dexamethasone; PCL, plasma cell leukemia; PR, partial response; (s)CR, (stringent) complete response; SD, stable disease; TLS, tumor lysis syndrome; VAD, vincristine-doxorubicin-dexamethasone; VGPR, very good partial response; wk, weeks*.

## Published Clinical Trials

### CD138

CD138 or syndecan 1, a member of the syndecan family of type I transmembrane proteoglycans, is highly expressed on the MM cell surface and is directly involved in disease progression (38). The latter works through binding to a proliferation-inducing ligand (APRIL), a survival factor (39), and cell proliferation-inducing growth factors (40). Interestingly, the expression of CD138 on MM cells of patients in relapse or with progressive disease is more pronounced than that on MM cells of newly diagnosed patients (38). Previous pre-clinical studies with NK cells expressing an anti-CD138 CAR showed potent anti-myeloma activity both *in vitro* and *in vivo* (41). Therefore, CD138 is a very attractive target for anti-MM therapy.

As shown in Table 2, one report recorded the use of anti-CD138 CAR-T cells in a patient with refractory MM with extramedullary involvement. Here, the administration of 1.5 × 10^8^ CAR-T cells led to partial response (PR) (31). A pilot clinical trial (ClinicalTrials.gov identifier, NCT01886976) reported the results of five patients with refractory and relapsed MM, pre-treated with chemotherapy and stem cell transplantation, who received an average dose of 0.756 × 10^7^ cells/kg of autologous CD138 CAR-T cells (Table 2) (32). All patients underwent a bone marrow examination, demonstrating CD138 expression in aspirates, and by biopsy. The *CAR* gene was continuously observed in the patients' blood for at least 4 weeks, and high levels of CAR-T cells were detected in the bone marrow at the first 2 months. Stable disease (SD) was achieved in four patients, ranging from 3 to 7 months, whereas the fifth patient progressed, even though CAR-T cells could be detected in the bone marrow for 90 days.

Although promising, CD138-targeted CARs should still be used with caution owing to the broad expression of CD138 in human tissues, including epithelial cells. For example, treatment with BT062, an antibody-drug conjugate directed against CD138, resulted in skin and/or mucosal toxicity (42). Nevertheless, pre-clinical work by Sun et al. has shown that CD138 CAR-T cells are safe and lack activity against normal epithelial cells (43). Like BCMA, CD138 can be shed from the MM cell surface, a possible escape route disrupting the effector functions of CD138-targeted immune cells (44). This underlines the importance of combining CD138 CAR-T cells with other CAR target antigens. Based on a search of the ClinicalTrials.gov registry using the search terms “multiple myeloma” and “chimeric antigen receptor” or “CAR” (final date of search May 1, 2019), numerous studies of CD138-targeted CAR-T cell therapy in combination with other CARs are ongoing or planned (NCT03196414, NCT03473496, NCT03271632). Apart from the above-mentioned NCT01886976 trial, only one other study could be identified in which CD138 CAR-T cells were used as stand-alone therapy (NCT03672318).

### CD19

Most myeloma cells resemble fully differentiated plasma cells and are CD19-negative. There is, however, a small subset of CD19-positive myeloma cells that are more pre-mature and have drug-resistant and disease-promoting qualities (45, 46). Targeting these MM stem cell-like cells could be of interest. In general, low expression of CD19 appears to be more common on MM cells than previously thought, correlating with poor survival (47, 48).

Garfall et al. conducted a pilot clinical trial (NCT02135406) of CD19 CAR-T cell therapy (CTL019) involving 10 MM patients with a progression-free survival of <1 year after their first stem cell transplantation (Table 2) (33). Patients were treated with a combination of high-dose melphalan, a second autologous stem cell transplantation (ASCT), and 1–5 × 10^7^ CTL019 cells (administered ~2 weeks post-ASCT). A case report was first published for one patient with a minimal residual disease-negative complete response (CR), persisting up to 12 months after CTL019 infusion (47). When looking at the complete dataset, 6 out of 10 patients experienced a very good partial response (VGPR) at day 100 post-transplant, and an additional two patients had a PR (33). The same group is also conducting a phase II clinical trial (NCT02794246) in which high-risk MM patients will receive CD19 CAR-T cells in the maintenance setting ~60 days after first-line ASCT.

### Combining CD19 and BCMA

CD19-specific CAR-T cells have also been used in combination with BCMA-targeted CAR-T cells, both in the relapsed/refractory setting (34) and in a newly diagnosed setting (35). Yan et al. reported on eight patients with relapsed/refractory MM; all patients experienced CAR-T cell-related cytokine release syndrome (CRS) but no neurological toxicity (Table 2) (34). Among the five patients with sufficiently long follow-up to evaluate for clinical response, one went into CR, one into VGPR, and two into PR (Table 2).

The same group also evaluated the safety and efficacy of combined CD19/BCMA CAR-T cell infusion in 10 patients with newly diagnosed MM after standard induction chemotherapy and ASCT (35). The study, registered with ClinicalTrials.gov under number NCT03455972, showed that CAR-T cells can be used as post-remission therapy to deepen the clinical response; of the four patients who were only in PR after transplantation, three went into VGPR following CAR-T cell administration and one obtained a CR. Toxicities, which included CRS in all patients, were mild and manageable.

Several other groups are also currently conducting clinical studies of the combination of CD19 and BCMA-targeted CAR-T cells (NCT03549442, NCT03706547, NCT03767725). The study by Garfall at University of Pennsylvania (NCT03549442) involves a randomization between BCMA CAR-T cells alone vs. the combination of BCMA CAR-T cells and CD19 CAR-T cells. The randomization phase of this trial aims to assess the value of BCMA ± CD19 CAR-T cell infusions as consolidation therapy in high-risk MM patients responding to frontline treatment. The combination of CD19 CAR-modified cells together with CD138 CAR-engineered immune effector cells is being evaluated in a pre-clinical context (49). Clinical trials of the latter combination are being awaited.

### Natural Killer Group 2, Member D (NKG2D) Ligands

The activating cell surface receptor NKG2D is commonly found on effector lymphocytes, including NK cells, CD8^+^ T cells, NK/T cells, and γδ T cells. Its ligands include major histocompatibility complex class I polypeptide-related sequence A/B and UL16 binding protein 1–6 (50). Under physiological conditions, tissues do not express NKG2D ligands on their surface. In contrast, neoplastic transformation will induce the upregulation of NKG2D ligands, including that in MM (51, 52).

Baumeister et al. constructed a CAR that targets multiple NKG2D ligands and performed a first-in human phase 1 clinical trial (36). They included five patients with relapsed/refractory progressive MM and seven patients with acute myeloid leukemia/myelodysplastic syndrome. None of the patients experienced CRS or neurotoxicity. NKG2D CAR-T cell persistence was limited and no objective clinical responses were observed (Table 2).

It is unclear whether or not this treatment failure was due to the fact that no lymphodepleting chemotherapy was used prior to CAR-T cell infusion (Table 2). Indeed, lymphodepletion appears to be important for CAR-T cell engraftment, and for clinical efficacy (53, 54). The fact that a first-generation CAR construct (i.e., without intracellular costimulatory domain) was used, may also have contributed to the lack of response. Another possible explanation for the absence of clinical activity may be the cell type that was used for CAR-T cell therapy. Pre-clinical work by the group of Leivas et al. revealed that only NKG2D CAR-transduced NK cells, but not T cells, are capable of killing MM cells and halting MM growth (55).

### Immunoglobulin Light Chains

Because impaired humoral immunity (i.e., B-cell depletion and profound hypogammaglobulinemia) is a well-known consequence of CD19-directed CAR-T cell therapy and CAR persistence, a more selective construct, sparing some B cells and hence partially preserving humoral immunity, may have improved applications. Mature B cells express either κ or λ light chains, but not both; thus, one of the two subsets can be targeted, leaving the other subset alone. Hence, this concept could be used to kill monoclonal MM cells expressing a certain type of light chain but not normal B cells expressing the reciprocal type of light chain. However, it should be noted that plasma cells generally do not express immunoglobulins on their surface, but secrete them into the bloodstream. Cases of MM-propagating cells expressing surface immunoglobulins have nevertheless been reported (56). Another possible drawback is that most patients who are candidates for CAR-T cell therapy already have B cell depletion at baseline due to previous therapies, making the evaluation of selective light chain therapy difficult.

Ramos et al. created the κ.CAR, a CAR construct specific for the κ light chain (37). In a phase 1 trial (NCT00881920), seven patients with MM and nine patients with non-Hodgkin lymphoma were included. These patients had heterogeneous prior therapy histories and salvage chemotherapies. In the seven patients with MM, no objective responses were observed (Table 2). One patient maintained stable minimal residual disease for 17 months; another patient maintained SD for 2 years, and two other patients exhibited transient SD (Table 2). CAR-T cell infusion was repeated in one patient after 1.5 years (after conventional therapy), which again led to transient SD. In the other three patients, no response to CAR-T cell therapy was documented. No severe CRS was observed, and no other complications were described (37).

## Ongoing Clinical Trials

### CD38

CD38 has been shown to be a promising target for the treatment of MM, considering the established clinical efficacy of anti-CD38 monoclonal antibodies, i.e., daratumumab (57). Unfortunately, CD38 is not only highly expressed on myeloma cells but also expressed at an intermediate level on hematopoietic cells, creating a real risk for on-target, off-tumor toxicity (58). For example, daratumumab has been shown to deplete NK cells, known to express CD38, in MM patients (57).

This potential of on-target, off-tumor toxicity can be a stumbling block to clinical implementation of CD38-directed CAR-T cell therapies. One of the strategies to circumvent this problem, involves the use of CARs with single chain variable fragments (scFvs) of lower affinity, generated through “light-chain exchange technology.” These low-affinity CD38 CAR-T cells are able to kill CD38^high^ MM cells, while having no effect on the viability of CD38^low^ healthy cells, as validated both *in vitro* and *in vivo* (59). A similar effect has been observed with CARs based on a CD38 nanobody instead of a scFv derived from a CD38 monoclonal antibody. Such CD38 nanobody-based CAR-T cells displayed potent cytotoxicity toward MM cells but only limited toxicity toward CD38-expressing normal hematopoietic cells (60).

Alternatively, researchers are also looking at selectively increasing the intensity of CD38 expression on the targeted tumor cells in order to maximize tumor-specific cytotoxicity and minimize on-target, off-tumor toxicity. CD38 can be upregulated on tumor cells by all-trans retinoic acid (61), or by the histone deacetylase inhibitor panobinostat (62). The combination of all-trans retinoic acid and CD38 CAR-T cell therapy has already been shown to be effective in a model of acute myeloid leukemia (61, 63).

Another strategy to control off-tumor effects is building CARs with a safety mechanism. Drent et al. produced a CD38 CAR based on the tetracycline-controlled Tet-on/off technology. *CAR* gene expression can be activated by the administration of low doses of the tetracycline doxycycline (64). The off-tumor effects produced by these CAR-T cells can be stopped within 24 h after doxycycline withdrawal. CAR expression can re-emerge upon rechallenge with doxycycline.

CD38 serves as the target antigen in several CAR-T cell clinical trials (65). In one study, CD38 CAR-T cells are used as monotherapy in patients with relapsed/refractory MM (NCT03464916). All other clinical trials are exploring potential combinations of CD38 CAR-T cells with other target antigens; with CD19 (NCT03125577), with BCMA (NCT03767751), with BCMA, CD138, or CD56 (NCT03473496, NCT03271632), and with BCMA and NY-ESO-1 (NCT03638206). Our group is currently investigating the possibility to simultaneously load lymphocytes with three different CARs, including CD38, CD19 and BCMA, by means of mRNA electroporation.

### Signaling Lymphocytic Activation (SLAM) Family Member 7 (SLAMF7)/CS1

SLAMF7, also known as CS1, is a member of the SLAM family of transmembrane receptors. First identified as a NK cell receptor, SLAMF7 also controls different functions of other immune cells, including subsets of CD4 and CD8 T cells, as well as B cells (66, 67). Moreover, SLAMF7 has been shown to be vital for phagocytosis of hematopoietic malignant cells by macrophages (68). No indications, however, have been found for SLAMF7 expression on other healthy cells and tissues. Because SLAMF7 is a robust marker of malignant plasma cells, it could be an interesting target for CAR-T cell therapy. Indeed, SLAMF7 expression has been observed on plasma cells of pre-malignant MM stages (i.e., MGUS and smoldering myeloma) and in newly diagnosed MM. SLAMF7 expression is further retained, even after several lines of therapy (69, 70).

A CAR construct was derived from the anti-SLAMF7 antibody elotuzumab and transduced into T cells from healthy donors and patients with MM (71). The generated CAR-T cells could efficiently kill MM tumor cell lines and primary MM cells. Like CD38, SLAMF7 is expressed on normal lymphocytes, including activated T cells, entailing a risk of CAR-T cell fratricide (71, 72). Indeed, it was confirmed that SLAMF7 CAR-T cells also killed healthy lymphocytes, but only those with high expression of SLAMF7. Lymphocytes with low expression of SLAMF7 were spared. This is very interesting considering that SLAMF7 CAR-T cells adopted a SLAMF7^low^ phenotype while in culture, ruling out problems due to CAR-T cell fratricide. Another strategy to decrease the risk of CAR-T cell fratricide involves the use of the transcription activator-like effector nuclease (TALEN) gene-editing technology during CAR-T cell manufacturing (72). Such TALEN-edited CAR-T cells no longer express endogenous SLAMF7 and are thus resistant to SLAMF7-driven CAR-T cell fratricide.

Currently, ongoing research focuses on identifying the optimal costimulatory moiety for the SLAMF7 CAR construct (i.e., 4-1BB or CD28) (71), the optimal lymphocyte source (i.e., autologous or allogeneic) (73), and the optimal cell type (i.e., T cells or NK cells) (8, 74). Pre-clinical work is also investigating whether dual SLAMF7/BCMA CAR-engineered T cells are superior to CAR-T cells expressing a single CAR molecule (75). In addition, SLAMF7 CAR-T cells are being tested in combination with other myeloma drugs, such as lenalidomide (76) and daratumumab (77).

To the best of our knowledge, three SLAMF7/CS1-based CAR-T cell products have entered the clinical trial pipeline. One study will use autologous, memory-enriched T cells lentivirally transduced to express a SLAMF7/CS1 CAR construct (NCT03710421). This CAR construct contains a truncated EGFR (EGFRt) molecule, permitting depletion of the CAR-T cells in case of severe side effects by administration of the anti-EGFR monoclonal antibody cetuximab (78). The European Union (EU), through the Horizon2020 program, is supporting a phase I/II clinical trial of SLAMF7 CAR-T cell therapy in MM, known as the CARAMBA project (for more details, see https://www.caramba-cart.eu/). These CAR-T cells are also equipped with the EGFRt safety switch, but the particularity about this product is the non-viral, Sleeping Beauty transposon-based method to transfer the *CAR* gene into the T cells. Finally, an “off-the-shelf” SLAMF7/CS1-directed CAR-T cell product for MM has recently been approved for clinical trial use. The product, also called UCARTCS1, contains healthy, allogeneic T cells loaded with a SLAMF7/CS1 CAR. TALEN technology is used prior to *CAR* gene transfer to disrupt the endogenous TCR and SLAMF7 expression in order to prevent alloreactivity and fratricide, respectively (73).

### CD44v6

CD44, the major hyaluronan receptor, is expressed on hematological cancer cells and is thought to play a role in cancer initiation (79). Unfortunately, CD44 is also expressed on the surface of healthy cells. However, the expression of the CD44v6 isoform is more restricted and can frequently be detected on advanced, high-risk MM cells (80). Casucci et al. created an anti-CD44v6 CAR for the treatment of acute myeloid leukemia and MM (81). T cells activated with CD3/CD28 beads, IL-7, and IL-15 were transduced with the CD44v6 CAR, and displayed potent cytotoxic effects against MM. No effects were observed against normal hematopoietic stem cells and CD44v6^low^ keratinocytes; however, CD44v6 CAR-T cells did cause a reversible decrease in monocyte count. This side effect can be beneficial, since monocytes are the main cause of CRS (82). Nevertheless, to minimize the risk of toxicity, safety switches under the form of suicide genes (i.e., thymidine kinase gene, or inducible caspase 9 gene) were incorporated (81). The same group also incorporated an extracellular spacer from the nerve-growth-factor receptor (NGFR) into the CD44v6 CAR construct. Using anti-NGFR immunomagnetic beads, the CAR-T cell product could be highly enriched for CD44v6 CAR-expressing T cells. This method opens up the possibility to purify T cells expressing the CAR and to omit the non-transduced cells (83). The EURE-CART project, supported by the EU Horizon 2020 program, involves a phase I/IIa clinical trial to determine the safety and efficacy of CD44v6 CAR-T cell therapy in patients with acute myeloid leukemia and MM (for more details, see https://www.eure-cart.eu/) (84).

### CD56

CD56 expression is found on a broad range of cells, including NK cells and other immune effector cells (85–87). Although it is not expressed on healthy plasma cells, CD56 is frequently expressed on MM cells (88). Lorvotuzumab mertansine, an antibody-drug conjugate against CD56, has recently been tested in a dose-escalation phase 1 clinical trial of 37 patients with relapsed MM (89). Treatment was well-tolerated, and some early signs of anti-MM activity were observed, strengthening further investigations of CD56 as a target in MM. One report described a CD56-directed CAR-T cell therapy with potent antimyeloma activity (90). However, no further results were published. The clinical benefits and potential toxicities of targeting CD56 are not known, but caution should be exercised owing to the broad expression of CD56. Indeed, depletion of CD56-positive immune effectors cells by treatment with lorvotuzumab entails a risk of infection; infection-related deaths were observed with this antibody-drug conjugate in a clinical trial of patients with small cell lung cancer (87, 91). As discussed above, CD56—in combination with other target antigens—has been adopted in two CAR-T cell clinical trial protocols for MM (NCT03473496, NCT03271632).

### G Protein-Coupled Receptor Class C Group 5 Member D (GPRC5D)

GPRC5D, a myeloma cell surface antigen whose precise function remains to be defined, has recently been proposed as an attractive candidate for anti-myeloma CAR-T cell therapy (92). The antigen is expressed on CD138-positive MM cells; it also expressed in the hair follicle, a potentially immune-privileged site therapy limiting the risk for on-target, off-tumor toxicity. Most interestingly, the expression of GPRC5D is independent of BCMA. Hence, GPRC5D-targeted CAR-T cells could potentially rescue patients experiencing an antigen-loss relapse under BCMA-directed CAR-T cell therapy (92). This hypothesis has been confirmed in a murine BCMA antigen escape model (93) and has paved the way for the MCARH109 trial, a phase I clinical trial to evaluate GPRC5D CAR-T cell therapy in relapsed/refractory MM patients including those who have received prior BCMA-directed therapies (92).

### Transmembrane Activator and CAML Interactor (TACI)

Like BCMA, TACI is a member of the tumor necrosis factor receptor superfamily that is expressed on malignant plasma cells, albeit usually at lower levels (94). APRIL is a naturally occurring ligand for both BCMA and TACI; as discussed above, CD138 is required as a co-receptor for binding of APRIL to TACI (95). APRIL-based CAR-T cells have been developed for dual targeting of BCMA and TACI on myeloma cells (94, 96), and clinical studies have been initiated (ClinicalTrials.gov identifier NCT03287804). Interestingly, pre-clinical work by Lee et al. has shown that APRIL-based CAR-T cells can kill BCMA^+^TACI^+^ as well as BCMA^−^TACI^+^ myeloma cells. This indicates that APRIL CAR-T cell therapy can maintain tumor control in case of BCMA downregulation, which is a well-described tumor escape mechanism in BCMA-directed CAR-T cell studies (94). Furthermore, it was recently shown that TACI is also expressed on regulatory T (T_reg_) cells in patients with MM. As such, APRIL-based CAR-T cells have the potential not only of targeting MM cells directly, but also indirectly by suppressing T_reg_ cells (97).

### Lewis Y

The Lewis Y (LeY) antigen is a carbohydrate antigen that is overexpressed on a variety of tumor cells, including MM cells. LeY expression is found in approximately 50% of MM cases (98). The antigen is related to the Lewis blood group antigen system, but not expressed on the red blood cell membrane. Overall, LeY has limited expression in normal cells and tissues, and no evidence of on-target, off-tumor toxicity was found with anti-LeY CAR-T cells in pre-clinical studies (99). A phase I clinical trial of anti-LeY CAR-T cell therapy for hematological malignancies (including MM) was registered with ClinicalTrials.gov already in 2012 (NCT01716364), but the status of this study is unknown and—to the best of our knowledge—no results have been published yet.

### New York Esophageal Squamous Cell Carcinoma 1 (NY-ESO-1)

One of the main limitations of CAR-T cell therapy is that it is only applicable to cell surface antigens, but not to intracellular oncoproteins. Such antigens are usually expressed in the context of HLA molecules and recognized by the T-cell receptor (TCR). NY-ESO-1 is an example of an intracellular oncoprotein that serves as target for TCR-engineered T cell immunotherapy in MM (100). TCR-mimetic CARs recognizing the NY-ESO-1/HLA complex, have been developed (101, 102). In a mouse model of NY-ESO-1/HLA-A2^+^ MM, NY-ESO-1-directed CAR-T cells were capable of delaying MM growth (102). The anti-myeloma activity could by further improved by co-infusion of T cells that were genetically engineered to express the NY-ESO-1 antigen and membrane-bound IL-15. These NY-ESO-1/IL-15^+^ T cells served as antigen-presenting cells and were found to improve the persistence of NY-ESO-1 CAR-T cells with a memory phenotype (102). One NY-ESO-1-directed CAR-T cell clinical trial (combined with other target antigens) for MM has been registered with ClinicalTrials.gov (NCT03638206).

## Preclinical Studies

### CD229

SLAMF3, also known as Ly9 or CD229, is another receptor of the SLAM family. It has a homogeneous expression on MM cells, which is stable regardless of the disease stage and exposure to different treatments, and it plays an essential role in the survival of MM cells (103–106). Moreover, CD19^−^CD138^−^ MM cells, which represent a quiescent, drug-resistant myeloma-propagating cell population (107), are highly positive for CD229 (104). This implies that CD229 CAR-T cells would be able to eradicate both the bulk of MM cells, and chemotherapy-resistant minimal residual disease. The first CD229 CAR-T cell construct was generated by the group of Atanackovic et al. (108). CD229 CAR-T cells demonstrated a strong cytotoxic activity against CD229-positive myeloma cell lines, with only minor activity against B cells and resting T cells. The most interesting finding came from a mouse model engrafted with luciferase-expressing U266 MM cells. Whereas, mice treated with CD19 CAR-T cells or phosphate-buffered saline still showed a clearly detectable bioluminescence signal after 18 days, the CD229 CAR-T cells had completely eradicated the MM cells (108). To the best of our knowledge, a clinical trial with CD229 CAR-T cells in MM has not yet been registered.

### Integrin β7

Because finding a myeloma-specific target antigen is quite difficult, some recent research has focused on non-cancer-specific epitopes that become specific after post-translational events, such as glycosylation or conformational changes. As such, integrin β7 has been identified as a potential target for MM by screening more than 10,000 hybridomas against MM tumor cells (109). MMG49, a monoclonal antibody identified from that screening assay as having the highest potential, is able to specifically recognize cancer-specific conformation of integrin β7 and a small fraction of CD19-positive B cells. Further studies showed that MMG49 was directed at a configuration-sensitive epitope of integrin β7, targeting the activated state that is highly expressed by MM cells. *In vitro*, MMG49 CAR-T cells were able to proliferate, secrete the immunostimulatory cytokines interferon-γ and IL-2, and efficiently eradicate MM cells. There was no indication of myeloma cells escaping therapy. Healthy hematopoietic cells were left untouched, even when integrin β7 was activated. After humanizing the mouse-derived scFv, MMG49 CAR-T cells will be tested in clinical trials (109).

### CD70

One of the first CARs developed for MM was directed against the tumor necrosis factor family member CD70 (CD27L), which plays a role in plasma cell differentiation (110). Shaffer et al. constructed a CD70 CAR with an antigen-binding domain derived from CD27 and fused to the intracellular domain of the CD3ζ chain (111). In this way, the CAR-T cells were able the kill CD70-positive MM cells and, at the same time, take advantage of CD27/CD70 co-stimulation, leading to enhanced T cell survival. In a murine xenograft model, CD70-specific CAR-T cells led to sustained regression of established lymphoma. The low and variable expression of CD70 on myeloma cells limits the use of CD70-directed CAR-T cells in MM (112).

### CD1d

The MHC class I-like molecule CD1d is highly expressed on pre-malignant and early MM cells, followed by a gradual decline in expression level with disease progression (113). The immune cells known to respond to glycolipids presented in the context of CD1d, are NK/T cells. Taking advantage of the intrinsic characteristics of NK/T cells, CD19 CAR-NK/T cells are able to target both CD19 and CD1d on MM cells, resulting in a reinforced anti-tumor effect as compared to CD19 CAR-T cells (10). Strengthening this therapeutic avenue is the low cytotoxicity of the CD19 CAR-NK/T cells against monocytes, the highest CD1d-expressing blood cells (113). Hence, NK/T cells are interesting effector cells for CAR-based cellular immunotherapy against CD1d-expressing malignant cells, including (early-stage) MM. Certain drugs, such as EZH2 inhibitors and ATRA, are known to increase CD1d expression on the MM cell surface (10), opening up the perspective for combination therapy.

## Conclusions and Future Perspectives

While the experience with BCMA-targeted CAR-T cells has provided robust evidence for the high therapeutic potential of CAR-T cell therapy in MM, we must not lose sight of the fact that responses are often temporary and that half of the patients will have relapsed or progressed after 1 year (Table 1). One of the main reasons for these relapses is downregulation or loss of BCMA expression on the MM surface. The exact mechanism for this downregulation is still unclear. Shedding of BCMA into the bloodstream is one possibility. Moreover, it was recently elucidated that the downregulation of BCMA can also be the result of CAR-T cell-induced trogocytosis, a process in which the BCMA molecule is transferred from the tumor cell to the CAR-T cell surface. The CAR-T cells then become BCMA-positive and will start recognizing each other, leading to CAR-T cell fratricide (29). Whether or not CAR-T cell therapy will revolutionize the treatment of MM will largely depend on how we will be able to deal with this problem of antigen escape. The answer to this question probably lies in the identification of additional antigens that can be targeted in combination with BCMA.

One potential strategy involves the combined infusion of two (or more) CAR-T cell products, such as BCMA CAR-T cells and CD19 CAR-T cells. The goal here is to eradicate not only the bulk myeloma cells (BCMA-positive) but also the small reservoir of myeloma “stem cells” (CD19-positive), thereby increasing the likelihood of achieving a durable clinical response. As discussed above, the combination of BCMA and CD19 CAR-T cells has already proven to be highly clinically efficacious (34, 35). Nevertheless, the results of an ongoing randomized study comparing BCMA/CD19 CAR-T cells with BCMA CAR-T cells alone (NCT03549442) need to be awaited in order to draw conclusions about the potential superiority of this combinatorial approach.

Instead of co-administering two separate CAR-T cell products, compound CAR-T cells are gaining increasing attention (114). Compound CAR-T cells are T cells expressing two (or more) different CARs (75). The idea is to target multiple antigens at the same time in order the overcome the limitation of loss of one particular antigen (114). Chen et al. have developed a compound CAR-T cell co-expressing a BCMA and SLAMF7/CS1 CAR. The authors found that BCMA CAR-T cells alone left a small population of (SLAMF7/CS1^+^) myeloma cells whereas the compound CAR-T cells effectively depleted both the BCMA^+^ and SLAMF7/CS1^+^ cells (75).

The strategy proposed by Smith et al. deserves further consideration (93). In a murine BCMA CAR-T cell model, the authors have elegantly shown that BCMA loss-mediated relapses can be avoided by subsequent targeting of a different myeloma surface antigen (i.e., GPRC5D) (93). The drawback of this approach is the need to manufacture different batches of CAR-T cells and further increasing costs. Moreover, in a recently published mouse model of CD19/CD22 CAR-T cell therapy, it was shown that concomitant targeting was more effective than the sequential approach at preventing antigen escape (29).

The same study also indicated that the choice of costimulatory domain might be critical for therapeutic success in combinatorial CAR-T cell approaches (29). For example, incorporation of the CD28 costimulatory domain in the CD19 CAR construct and 4-1BB in the CD22 CAR construct proved to be best combination of costimulatory domains in terms of synergistic activity. This combination was also the most effective in case of diminished expression of CD19 by the target cells. Although it remains to be examined whether these results are extrapolatable to the myeloma CAR-T cell field, the study clearly highlights the importance of rational CAR design especially in combination CAR-T cell therapy.

In conclusion, our knowledge of the mechanisms responsible for relapses following BCMA-CAR-T cell therapy is rapidly expanding. Besides tumor antigen downregulation or loss, other contributors of relapse, such as the development of anti-CAR-T antibodies, insufficient CAR-T cell persistence, or perhaps even more importantly, T cell exhaustion, are important topics of research (53). This increasing knowledge of the mechanisms of relapse, along with the identification of novel CAR target antigens, increases the likelihood that the full therapeutic potential of CAR-T cell therapy for MM will be unleashed in the near future.

## Author Contributions

MT, GR, HV, and SA reviewed the literature, participated in the design of the manuscript, and wrote the paper. YW, DC-D, VV, YC, ZB, and FL revised the manuscript substantially for important intellectual content.

### Conflict of Interest Statement

The authors declare that the research was conducted in the absence of any commercial or financial relationships that could be construed as a potential conflict of interest.
